# Mutational analysis of dimeric linkers in peri- and cytoplasmic domains of histidine kinase DctB reveals their functional roles in signal transduction

**DOI:** 10.1098/rsob.140023

**Published:** 2014-06-04

**Authors:** Jiwei Liu, Jianguo Yang, Jin Wen, Yun Yang, Xiaolu Wei, Xiaodong Zhang, Yi-Ping Wang

**Affiliations:** 1 State Key Laboratory of Protein and Plant Gene Research, College of Life Sciences, Peking University, Beijing 100871, People's Republic of China; 2 Department of Life Sciences, Centre for Structural Biology, Imperial College London, London SW7 2AZ, UK

**Keywords:** DctB, two-component system, transmembrane signal transduction

## Abstract

Membrane-associated histidine kinases (HKs) in two-component systems respond to environmental stimuli by autophosphorylation and phospho-transfer. HK typically contains a periplasmic sensor domain that regulates the cytoplasmic kinase domain through a conserved cytoplasmic linker. How signal is transduced from the ligand-binding site across the membrane barrier remains unclear. Here, we analyse two linker regions of a typical HK, DctB. One region connects the first transmembrane helix with the periplasmic Per-ARNT-Sim domains, while the other one connects the second transmembrane helix with the cytoplasmic kinase domains. We identify a leucine residue in the first linker region to be essential for the signal transduction and for maintaining the delicate balance of the dimeric interface, which is key to its activities. We also show that the other linker, belonging to the S-helix coiled-coil family, plays essential roles in signal transduction inside the cell. Furthermore, by combining mutations with opposing activities in the two regions, we show that these two signalling transduction elements are integrated to produce a combined effect on the final activity of DctB.

## 2. Introduction

Two-component systems (TCSs) are widespread in all three kingdoms of life, especially in bacteria [1,2]. It is the major system for sensing and responding to environmental signals, such as virulence factors, antibiotics, quorum sensing, lipid modification, chemotaxis, osmolarity and nitrogen fixation [2,3]. Genome analysis shows that many bacteria have over 50 TCSs [2,4,5].

**Figure 1.**
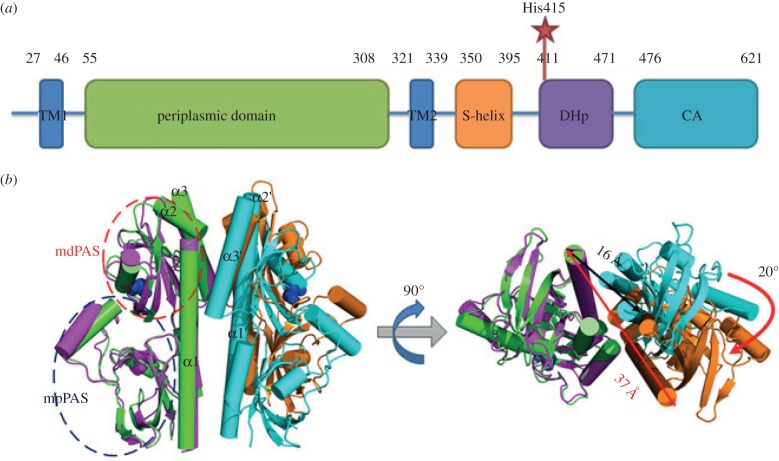
Overview of DctB HK. (*a*) Schematic diagram of DctB domain architecture. The residue numbers for each domain are annotated above. The histidine residue to be phosphorylated is marked as a star. (*b*) Ligand-binding induces conformational changes at the dimeric interface of DctBp. The succinate molecules are shown as blue spheres. The distance information of the N-termini of dimeric DctBp is shown in black for the *apo* state, and in red for the succinate-bound state. The two monomers of DctBp in the *apo* state (PDB ID: 3E4Q) are shown in green and cyan, and the two monomers in the succinate-bound state (PDB ID: 3E4O) are shown in magenta and orange. Each DctBp monomer consists of membrane distal (mdPAS) and membrane proximal PAS (mpPAS) domains. The mdPAS domain consists of the PAS core (red circled) and its flanking region α1-helix [7].

A typical TCS comprises a membrane-associated sensory histidine kinase (HK) and a cytoplasmic response regulator (RR) [4,5]. HKs are usually homodimeric proteins, and each monomer contains a periplasmic sensor flanked by two transmembrane helices, a cytoplasmic linker and a cytoplasmic kinase module [6]. The kinase module consists of a four helical bundle DHp domain and a catalytic CA domain (figure 1*a*). The CA domain binds and hydrolyses ATP to phosphorylate a conserved histidine residue on the DHp domain [8]. Subsequently, this phosphoryl group is transferred to the N-terminal asparagine residue of the RR, normally resulting in the activation of the RR [4,9,10]. Many RRs are transcription factors, regulating the transcription of relative genes in response to environmental signals [7].

In HK, signal perception normally occurs on its periplasmic sensor domain [11]. The Per-ARNT-Sim (PAS) domain is identified most frequently as the signal perception domain in HKs. The SMART database contains over 44 000 PAS domains in more than 29 000 proteins (December 2013), spanning all kingdoms of life. Most known PAS domains contain a PAS core and flanking regions [12]. The PAS core has a well-conserved tertiary structure of three to four α-helices flanking a central five-stranded anti-parallel β-sheet (figure 1*b*), which is essential for the ligand binding [12]. The flanking region usually adopts α-helical conformations [12–15] and serves as a linker between the PAS core and other parts within the larger protein [12]. It was also proposed that the signals originated upon ligand binding to the PAS core would be propagated through the flanking region of the PAS domain to other parts of the protein [12,16]. However, it is unclear through what mechanism the flanking region could transduce the signal.

Immediately following the transmembrane helix is a cytoplasmic linker region that is proposed to play key roles in transducing signals to activate the kinase domain. About three-quarters of all TCS HKs contain helical linkers in the cytoplasmic portion [17]. A conserved HAMP linker has been identified in some TCS HKs [18]. Structural and mutational analysis suggests an essential role of the HAMP linker in signal transduction from the environmental stimuli to the cytoplasmic kinase domains [11,19–24]. Recently, a novel linker family, termed S-helix (signalling helix), has been identified [25]. S-helix has been found in thousands of signalling proteins from bacteria to humans, separating diverse sensor domains from the enzymatic domains, such as in HKs, cNMP cyclases, PP2C phosphatases, AAA+ ATPases and diguanylate cyclases [25]. S-helix linkers are predicted to form dimeric coiled coils [25]. In yeast osmosensor Sln1p and bacterial nitrate sensor NarX proteins, the S-helix linkers were shown to play essential and conserved roles in the signal transduction from the sensor domains to the enzymatic domains [11,26,27].

Coiled coils are common structural features that usually consist of two or more α-helices forming helical bundles. The primary structure of a coiled coil is represented by heptad repeats denoted (abcdefg)*_n_*, where *n* is the number of repeats. Positions ‘a’ and ‘d’ are generally hydrophobic residues and they form the ‘knobs and holes’ at the buried core, responsible for the stability of the coiled-coil structure. Positions ‘e’ and ‘g’ are usually hydrophilic residues located on the outer rim of the coiled coil and their interactions further stabilize the coiled-coil structure [28].

DctB is a typical HK sensor, responsible for detecting the major energy and carbon sources C_4_-dicarboxylic acids (DCA) for nitrogen fixation in symbiotic rhizobium. In the presence of DCA, DctB phosphorylates its RR DctD, which in turn activates the transcription of the DCA-transporter-encoding gene *dctA* [29–31]. In particular, the *apo* form of DctB is at an inactive state (‘OFF’), whereas the ligand-binding induces an activated state (‘ON’) [7]. DctB detects stimuli with its periplasmic sensor domain (residues 55–308, DctBp) and transmits the signal to the cytoplasmic kinase module (residues 411–621, DctBk) (figure 1*a*). Previously, the homodimeric crystal structures of the *Sinorhizobium meliloti* DctB periplasmic domain in both *apo* and succinate-bound states have been determined. Each DctBp monomer is composed of two PAS domains. A long N-terminal α-helix (α1) links the membrane distal PAS (mdPAS) domain with the first transmembrane helix (figure 1*b*). Structural analysis reveals that succinate binding to the mdPAS domain induces conformational changes mainly at the dimeric level. In this case, each monomer moves as a rigid body, resulting in about a 20 Å distance increase between the two N-termini of DctBp as well as a 20° rotation along the axis perpendicular to the membrane plane (figure 1*b*) [7,13]. A salt bridge network among residues D89-K110–D89′-K110′ (’ indicates residues from the other monomer) is formed in the ‘ON’ conformation. Perturbing this network through mutations (such as K110D mutation) resulted in constitutively inactive DctB [7,13].

In this study, we investigated the key determinants for the transmembrane signal transduction in DctB. In the periplasmic side, we identified a single leucine residue (L71) on the dimeric interface of DctBp to be essential for maintaining both the ‘OFF’ state and the signal transduction. Mutagenesis analysis indicates that the integrity of this hydrophobic residue is key to the proper signal transduction. In addition, we show that a delicate balance of interactions at the dimeric interface and the ability to form and dissociate the dimer are essential for response to signal. In the cytoplasmic side, we identified a typical S-helix linker of about 40 amino acids in DctB [25]. Mutating a number of conserved residues in the S-helix linker resulted in hyperactive or inactive phenotypes of DctB, suggesting its essential roles in signal transduction. Combining mutations with opposing activities in the above two regions, we show that these two signalling transduction elements have a combined effect on the final activity of DctB.

## 3. Results

### 3.1. A delicate balance of the dimer interface is essential in controlling signal transduction

The signal perceived by DctBp was supposed to be transduced via the dimeric interface of DctBp [7,13]. The dimeric interface is composed of the membrane distal part and membrane proximal part [7,13]. Upon ligand binding, the membrane proximal dimeric interface dissociates while the membrane distal dimeric interface is still maintained (figure 1*b*). The dissociation of the membrane proximal dimeric interface is essential to induce changes that ultimately lead to the activation of the catalytic domain. In order to understand the molecular basis for the changes at the dimeric interface, we analysed key residues at the interface.

**Figure 2.**
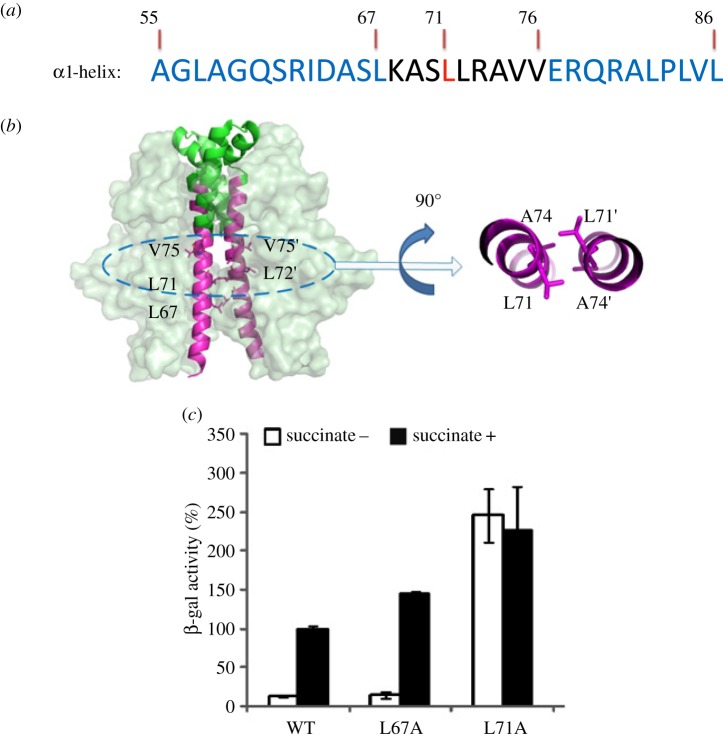
Analysis of the role of the α1-helix during signal transduction. (*a*) Sequence of α1-helix of DctBp. The position of residues is annotated above. The central region is coloured black with L71 highlighted in red. (*b*) Structure of α1-helices of DctBp in the *apo* state. The dimeric DctBp is shown as grey surface. The α1-helices are highlighted in magenta cartoon. The α2- and α3-helices are show in green cartoon. Hydrophobic residues of the α1-helices are shown as magenta sticks. Prime symbol (′) indicates the corresponding residue of the other monomer. The central region of α1-helices is enlarged to show the ‘knobs into holes’ interactions formed between A74 and L71. (*c*) *In vivo* activities of the DctB-carrying mutations on L67 and L71. All activity assays were performed in *E. coli* TP2339 (*crp-39, lacΔX74*) and the activities of wild-type DctB induced by 2 mM succinate is taken as 100%.

**Figure 3.**
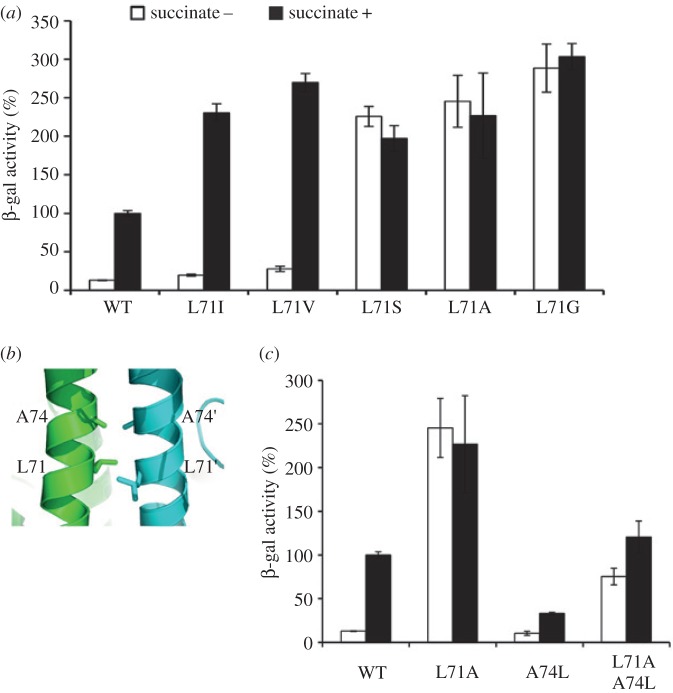
Mutational analysis of key residues controlling the dimeric interface of DctBp. (*a*) *In vivo* activities of the DctB-carrying mutations in L71. (*b*) The 74th residue is another packing position for α1–α1′ dimer. The two α1-helices are parallel between residues L71 and A74 in the *apo* state of DctBp. (*c*) *In vivo* activities of the DctB-carrying mutations on A74 or/and L71. All activity assays were performed as described in figure 2.

Previous structural data show that in the ‘OFF’ state, the membrane proximal dimeric interface is a coiled coil formed by two α-helices (residues 67–76 in α1 and α1′, figure 2*a*,*b*). The N-terminal part of the two α1-helices curve away from each other (residues 55–66), while the C-terminal part of the α1-helix (residues 77–86) is involved in interactions with other parts of DctBp (figure 2*a*,*b*). The middle section, containing residues 67–76, could therefore play crucial roles in the change from the ‘OFF’ to the ‘ON’ state. Specifically, three residues, L67, L71 and A74 appear to form the ‘knobs’ and ‘holes’ of the dimeric coiled coil (figure 2*b*), maintaining the ‘OFF’ state. SOCKET [32] analysis of DctBp *apo* structure (PDB: 3E4Q) did identify residues L71 and A74 (and only these two) participating in the classical ‘knobs into holes’ interactions (figures 2*b* and 3*b*). To investigate their roles further, we mutated these two leucine residues to alanine separately and analysed the activity of the mutant proteins in terms of their ability to activate transcription in the absence and presence of an inducing agent, succinate. The activities are measured using a reporter system on a plasmid with a DctB-regulated *dct*A promoter [7]. DctB-L67A acts similarly to wild-type proteins, while DctB-L71A is constitutively active and no longer responds to inducing signals (figure 2*c*), suggesting the importance of L71 in maintaining the ‘OFF’ state conformation.

To further investigate the roles of L71, we carried out additional mutagenesis analysis. Our results show that all small side chain variants, such as DctB-L71A, DctB-L71G and DctB-L71S, are constitutively active *in vivo* (figure 3*a*), and could not be further induced, even when the concentration of succinate was as high as 40 mM. On the other hand, two conservative hydrophobic changes, DctB-L71I and DctB-L71V, are inducible (figure 3*a*). These results suggest that the hydrophobic property of L71 is essential to maintain the ‘OFF’ conformation under non-inducing conditions. Interestingly, the activities of DctB-L71I and DctB-L71V are higher than that of the wild-type. However, the ratio between the activities of the induced and non-induced DctB (we define this as inducibility in table 1) is relatively similar, suggesting that the system is robust in its response to signals as long as residue 71 is hydrophobic (L, I or V).

**Table 1.** *In vivo* activities of DctB wild-type and mutants.

DctB constructs	activity (%)		inducibility
	0 mM succinate	2 mM succinate	
WT	13 ± 0	100 ± 4	8
WT^a^	14 ± 4	215 ± 78	15
L67A	15 ± 4	145 ± 2	10
L71I	20 ± 1	231 ± 11	12
L71V	28 ± 3	270 ± 12	10
L71S	226 ± 13	197 ± 17	1
L71A	245 ± 34	227 ± 55	1
L71G	289 ± 31	303 ± 17	1
L71C	42 ± 8	66 ± 15	2
L71C^a^	34 ± 4	633 ± 134	18
A74L	10 ± 2	33 ± 1	3
L71A + A74L	75 ± 10	120 ± 19	2
K110D	10 ± 2	17 ± 6	2
L71A/K110D	71 ± 17	75 ± 12	1
T366I	5 ± 3	13 ± 10	3
T366L	9 ± 1	20 ± 15	2
T366V	6 ± 3	28 ± 19	5
T366K	73 ± 6	153 ± 4	2
T366R	51 ± 17	104 ± 2	2
T366D	244 ± 29	215 ± 28	1
T366E	265 ± 31	226 ± 29	1
L71A-T366L	52 ± 15	56 ± 7	1
K110D/T366E	84 ± 18	83 ± 7	1

^a^Activity with 10 mM DTT.

Our data so far suggest that the hydrophobicity and size of the L71 side chain are important in maintaining the coiled-coil structure in the ‘OFF’ conformation. As A74 also contributes to the dimer interface (figures 2*b* and 3*b*), we questioned whether the size and hydrophobicity of residue 74 are also important. We mutated A74 to a leucine residue, and the results show that the activity of DctB-A74L remains low irrespective of the addition of the inducer (figure 3*c*), in contrast to L71A which stays constitutively active. These data suggest that a delicate balance of the dimer interface is required to maintain the ‘OFF’ state and to switch to the ‘ON’ state. When the interactions at the dimer interface are strengthened, as in A74L, the activity stays low irrespective of the inducing agent, implying that the protein is unable to switch to the ‘ON’ state. On the other hand, when the dimer interface is weakened, as in L71A, L71G and L71S, the protein switches to the activated state without inducing agent. Indeed, the combined double mutant DctB-L71A/A74L, which presumably results in a dimer interface that has properties in-between the single mutations, has a final activity that is in-between those of individual mutants (figure 3*c*).

### 3.2. The dissociation of the dimeric coiled coil α1–α1′ is essential in signal transduction

**Figure 4.**
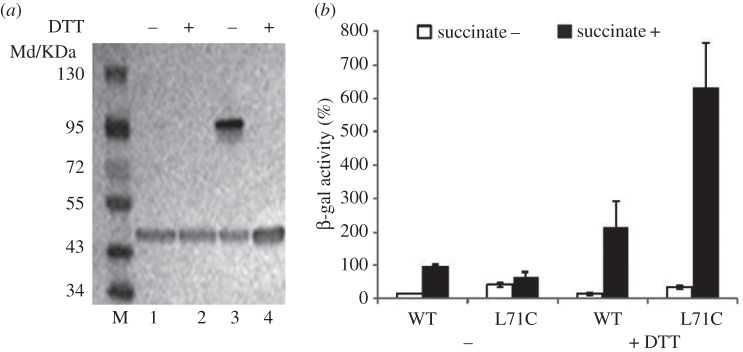
*In vitro* and *in vivo* analysis of disulfide bridge of L71C mutant. (*a*) *In vitro* analysis of disulfide bridge of DctB. Denatured His_6_-DctB_395_ under non-reduced (lane 1) or reduced (lane 2, 5 mM DTT) condition are pure monomers. Denatured His_6_-DctB_395_-L71C under non-reduced condition (lane 3) is mostly the dimer and could be reduced to pure monomers by 5 mM DTT (lane 4). (*b*) *In vivo* activities of the DctB-carrying cysteine mutations in L71. DTT 10 mM has been added to the media as indicated.

The above data show that a delicate balance at the dimer interface is essential for signal transduction in DctB. We reasoned that this is due to the required dissociation of the α1–α1′ coiled coil upon succinate binding in order to induce further downstream changes. To confirm this, we introduced a disulfide bridge by mutating L71 to a cysteine residue in order to covalently link the two α1– α1′-helices, artificially forbidding the dissociation of the α1–α1′ coiled-coil dimer. We expressed DctB without the kinase domain (residues 1–395, termed as DctB_395_) to confirm that this mutation indeed induces covalently linked dimers. SDS-PAGE analysis shows that approximately 70% of His_6_-DctB_395_-L71C forms covalently linked dimers (figure 4*a*). Similar results have also been observed in the full-length DctB (electronic supplementary material, figure S1). We then introduced the same mutation in our *in vivo* assays and probed the activities of full-length DctB-L71C. The results show that DctB-L71C has similar activities to that of wild-type in the absence of succinate, confirming that DctB-L71C is in the ‘OFF’ state. However, the inducibility is significantly reduced (figure 4*b* and table 1), confirming that DctB-L71C is defective in activation. Addition of the reducing agent dithiothreitol (DTT) recovered the activity of DctB-L71C to about three times of that of wild-type under the same condition (figure 4*b*). As a result, the inducibility of DctB-L71C is recovered to slightly higher than that of wild-type in the same reducing condition (table 1). These results further support the idea that a dissociable dimer interface is essential for signal transduction and induction.

### 3.3. Differential roles of L71 and the salt bridge network in signal transduction of DctB

**Figure 5.**
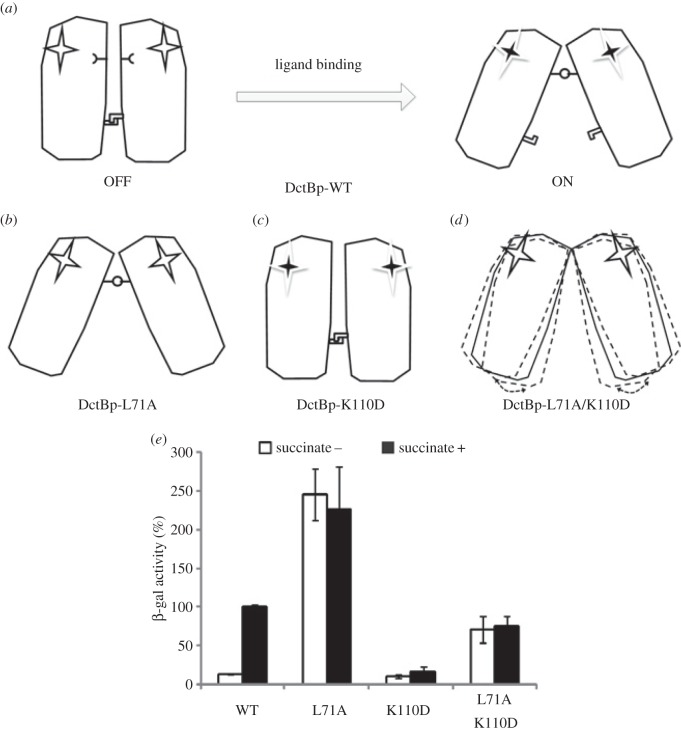
Differential role of L71–L71′ and salt bridge (D89-K110–D89′-K110′) in signal transduction of DctB. (*a*) Schematic of the mechanism of signal transduction of wild-type DctBp. Upon ligand binding, the dimeric interface of DctBp would be dissociated (L71–L71′), a salt bridge network (D89-K110–D89′-K110′) would form; 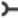 represents the D89-K110 residues and 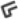 represents the L71 residue in each DctBp monomer, respectively. The open start represents the binding pocket of each DctBp monomer without a ligand bound, and the black star represents the binding pocket of each DctBp monomer with a ligand bound. (*b*) Schematic of the conformation of DctBp-L71A, staying in the ‘ON’ state. (*c*) Schematic of the conformation of DctBp-K110D, staying in the ‘OFF’ state. (*d*) Schematic of the conformation of DctBp-L71A/K110D. In the absence of both L71 and salt bridge, it is possible that the protein would adopt a conformation between the two extreme ‘OFF/ON’ conformations (solid line) or oscillate between them (dashed lines). (*e*) *In vivo* activities of DctB wild-type and mutants. All activity assays were performed as in figure 2.

Previous structural and biochemical analysis [7,13] revealed that a salt bridge network consisting of D89-K110–D89′-K100′ is essential for maintaining the ‘ON’ conformation (figure 5*a*). To investigate the interplay between the L71–L71′ and the salt bridge network in signal transduction, we constructed a double mutant that perturbs both ‘OFF’ (L71A) and ‘ON’ states (K110D) (figure 5*b*–*d*). Compared with single mutations, the double mutant has an activity somewhere in-between (figure 5*e*), in agreement with the idea that the ‘ON’ and ‘OFF’ conformations are stabilized by the salt bridge network and the hydrophobic interactions between the leucine residues, respectively. In the absence of one of the two stabilizing interactions, the conformation stabilized by the other one dominates (figure 5*b*,*c*). In the absence of both stabilizing interactions (as in the double mutants), it is possible that the protein would adopt a conformation between the ‘OFF’ and ‘ON’ conformations or would oscillate between them (figure 5*d*), with an average activity in-between the two (figure 5*e*). The inducibility is significantly reduced, suggesting that the system has lost its ability to respond to signal.

### 3.4. Characterization of the S-helix linker of DctB

**Figure 6.**
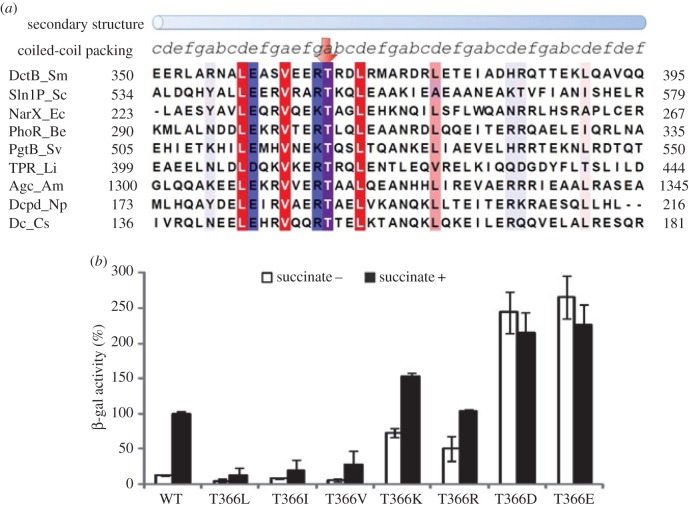
Characterization of the S-helix linker of DctB and its role in signal transduction. (*a*) Sequence properties of S-helix linker. Multiple sequence alignment of representatives of S-helix linker. Residues conserved in at least two-thirds of the 250 S-helix sequences are coloured according to hydrophobicity (red, hydrophobic; blue, hydrophilic; purple, polar). The prediction of secondary structure and coiled-coil registration of the DctB S-helix are annotated above the sequence. (*b*) *In vivo* activities of DctB with mutations at T366. All activity assays were performed as described in figure 2.

The signal perceived by DctBp would be transduced to the kinase module via a cytoplasmic linker. A PSI-BLAST search and sequence alignments indicate that the linker region of DctB is a typical S-helix linker, with members including Sln1P from *Saccharomyces cerevisiae* and NarX from *Escherichia coli* (figure 6*a*). Highly conserved residues include hydrophobic residues L358, V362, L369, L376, hydrophilic residues E359 and R365 and a polar residue T366 (figure 6*a*). Sequence analysis of the S-helix linker using coiled-coil prediction programs results in two different coiled-coil packing models, one continuous coiled coil and one with a discontinuity at residue V362 (figure 6*a* and the electronic supplementary material, figure S2) [28,33,34]. In either model, all hydrophobic residues mentioned above are predicted to be in an ‘a’ or ‘d’ position in the coiled coil (figure 6*a* and the electronic supplementary material, figure S2), serving as core residues to stabilize the dimeric coiled coil. To probe the importance of these conserved residues, we mutated L369 and L376 to A, R365 to E and T366 to either L (hydrophobic), R (positively charged) or E (negatively charged). Interestingly, L369A has little effect on its activity while L376A results in mainly inactive proteins (electronic supplementary material, figure S3), suggesting that these two leucine residues may play different roles. R365E mutation resulted in the loss of function (electronic supplementary material, figure S3), highlighting the potential importance of this positively charged residue. Importantly, T366, a conserved polar residue that is predicted to occupy an ‘e’ or ‘a’ position in the two different coiled-coil models, showed drastically different phenotypes depending on the type of mutations. When T366 is mutated to a hydrophobic L, I or V, the activity of DctB is significantly reduced; when it is mutated to a charged residue (either R, K or E, D), it becomes constitutively active (figure 6*b*). The mutagenesis results are consistent with the discontinuous coiled-coil model which places T366 in the position ‘a’ (figure 6*a* and the electronic supplementary material, figure S2). The polar residue T366 in the coiled coil therefore serves as a key modulator for signal transduction. Similarly to the periplasmic coiled-coil linker, a delicate balance of the cytoplasmic S-helix linker is thus also required for DctB's activity. It is also notable that although T366R and T366K have similar levels of induced activities as wild-type DctB, their uninduced activities have elevated significantly, which result in reduced inducibilities (table 1, about four times less than wild-type).

### 3.5. Periplasmic DctBp and cytoplasmic S-helix linker have combined effects on transmembrane signal transduction of DctB

Our data here show that both the flanking helix in periplasmic DctB and the S-helix linker in the cytoplasmic side can modulate signal transduction and both transducers are delicately balanced to allow a regulated response in signal transduction. To investigate the influence of these two elements in the signal transduction, we took advantage of the mutants available on both the periplasmic and the cytoplasmic sides.

**Figure 7.**
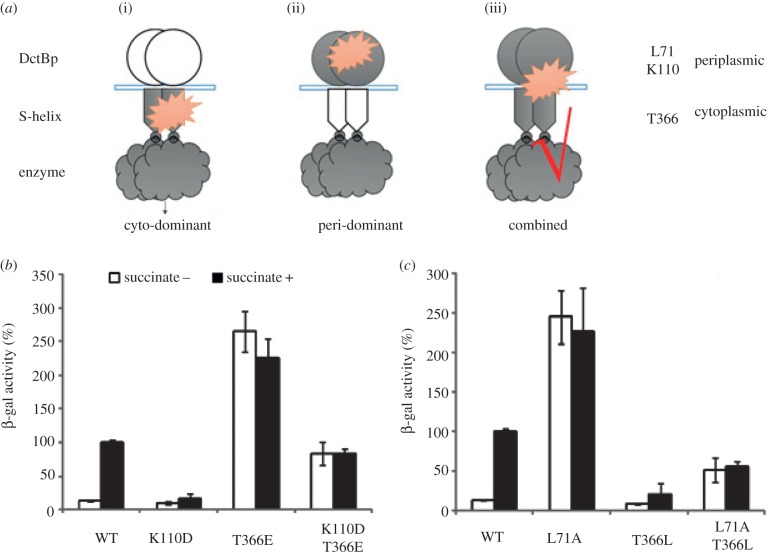
Analysis of double mutations in both DctBp and S-helix linker. (*a*) Three different relationships between peri- and cytoplasmic domains during transmembrane signal transduction. (*b*) *In vivo* activities of the DctB wild-type and mutants. All activity assays were performed as described in figure 2.

We constructed double mutants that are dominantly negative in the periplasmic domain and constitutively active in the cytoplasmic domain or *vice versa*. For example, the mutation K110D from DctBp, which resulted in a significant loss of activity, was combined with the mutation T366E from the S-helix linker, which results in hyperactive protein (figure 7). The hyperactive mutation from the DctBp (L71A) was combined with the defective mutant from the S-helix (T366L) (figure 7). The activities of these double mutations are in-between the activities of single mutants (figure 7*b*,*c*), suggesting that defects caused by one domain can be rescued by the other to some extent. These results show that both periplasmic and cytosolic signal transduction elements are important and the final activity is a result of a combined signal transduction process (figure 7*a*). Indeed, the activities suggest that both signal transduction elements act as amplifiers arranged sequentially and the final activity is thus an integrated result of both amplifiers.

## 4. Discussion

The cell membrane is considered as the biggest barrier for signals to be transmitted into a cell. Transmembrane HKs and their RRs play crucial roles in signal transduction in bacteria and eukaryotes [5]. In this report, through mutagenesis aided by previous structural work, we have identified a leucine residue, L71, that is essential for maintaining the *apo* ‘OFF’ state of DctBp. Interestingly, adding one extra hydrophobic residue by mutating A74 to leucine results in the constitutively ‘OFF’ state, inhibiting the signalling process. These data suggest that a delicate balance of the interactions at the dimer interface is required to maintain DctBp in the compact dimer conformation in the absence of ligand, but to enable the dimer to dissociate upon ligand binding. This notion is further supported by the *in vivo* analysis of DctB-L71C. Results show that by covalently linking the two monomers, the signalling process is inhibited. This process can be restored when the disulfide bridge is disrupted under reducing conditions (figure 4). Interestingly, DTT has an additive effect with succinate, even with wild-type protein (figure 4*b*). No clear binding between DTT and DctBp has been observed (electronic supplementary material, figure S4). The exact reasons for this enhanced activity are unclear, but it is unlikely to be due to unspecific binding of DTT to the protein as DTT has no effects in the absence of succinate (table 1).

It is interesting to note that although residue L67 is predicted to be at position ‘d’ in the dimeric α1–α1′ coiled coil, DctB-L67A mutation did not result in significant changes compared with the changes of DctB-L71A (figure 2*c*). Structural analysis shows that at L67, the two α1-helices start to curve away from each other, and L67 and L67′ are only loosely associated at the DctBp dimeric interface (electronic supplementary material, figure S5 and figure 2*b*). Consequently, only leucine (L71–L71′) and alanine (A74–A74′) pairs act as ‘knobs and holes’ to stabilize the central region of the dimeric α1–α1′ coiled coil.

Many HKs contain more than one PAS domain, acting as the sensor domains [12,35]. Previous studies suggested that the interactions between tandem PAS domains could potentially affect the signal amplification/integration [36]. In DctB, the linker between the tandem PAS domains is α5 [7,13]. Structural analysis suggested that α5 possibly interacts with α1 to form a four helical bundle at the dimer interface in the ‘OFF’ state (electronic supplementary material, figure S5). It is therefore possible that α5 can influence the dimerization of α1–α1′ and hence affect the ligand-induced activation. Signals detected by the mdPAS domain of DctBp could be possibly integrated/amplified with the membrane proximal PAS (mpPAS) domain through α5 via torque forces [36]. The changes in α5 could subsequently affect the dimerization of α1–α1′ via interactions between α5 and α1.

The oligomerization and dissociation of coiled-coil structures have been used as a means to regulate biological functions [37–39]. Dissociating a typical leucine zipper (with multiple pairs of ‘knobs’ and ‘holes’) is an energy-demanding process [39,40]. In the dimeric coiled-coil structure of α1–α1′, only a single leucine pair accompanied by an alanine pair act as the ‘knobs’ and ‘holes’ and no other potential hydrophilic interactions between residues at ‘e’ and ‘g’ positions have been detected in the crystal structures. Clearly, the system shown here is much weaker than the classical coiled coil, enabling it to dissociate readily. The hydrophobic strength of the α1–α1′ coiled-coil dimer is therefore carefully balanced to regulate the dimeric states of DctBp upon ligand binding.

It is widely accepted that intact HK is a dimer and the major dimerization determinants are the DHp domains, which exist as dimers in solution. Functional studies show that the dimerization of DHp is essential for the kinase activities. However, most sensor domains of HK, especially those consisting of PAS domains, are monomers or weak dimers in solution [35]. The membranes could provide additional restrictions to strengthen the dimer of the sensor domains. However, it is possible that the intrinsic weak dimer of HK domains (such as the sensor domains) has evolved for a specific signal transduction system. From the data here, we observe that the conformational changes in the dimeric level of DctBp are essential for the signal transduction. It is thus possible that the intrinsic weak dimerization of the sensor domains is necessary to maintain an appropriate response to the environment in other HKs as well.

For most membrane-associated HKs in TCS, the signal perceived in the periplasmic domain is transmitted across the membrane to the cytoplasmic kinase module via a conserved linker [5]. The mutagenesis analysis of conserved residues in the S-helix highlights the importance of this region and the potential functional and structural roles played by these conserved residues. Particularly interesting is T366, which displays opposing effects when mutated to hydrophobic or charged residues, pinpointing to a pivotal role in controlling the switch from ‘OFF’ to ‘ON’ conformations. Bioinformatics analysis suggests that the S-helix has a large propensity to form a dimeric coiled coil. These conserved residues could play important roles in the dimer interface, with T366 situated at pivotal points for the conformational switches during signal transduction. The mutagenesis results of T366 showed that when T366 is mutated to a hydrophobic residue, the protein becomes mainly inactive, while when T366 is mutated to a charged residue, it becomes hyperactive. These results suggest that the S-helix coiled coil also modulates the signal transduction and the activation of DctB. The predicted ‘a’ position of T366 implies that a hydrophobic residue would strengthen the coiled coil, while a charged residue would significantly weaken the coiled coil. The results of T366 in figure 6 are therefore in agreement with the idea that the delicate balance of the dimeric coiled coil formed by the S-helix serves as another modulator for the signal transduction. When the coiled coil is too stable as in T366V, T366I and T366L, the protein is in the ‘OFF’ state irrespective of inducing signals; when the coiled coil is weakened as in T366K, T366R, T366D and T366E, the protein is constitutively in the ‘ON’ state.

It is interesting to note that the DcuS from *E. coli*, which also detects DCA signals, uses a cytoplasmic PAS domain instead of S-helix to transduce the signal in the cytoplasmic regions [41]. Monzel *et al*. [41] suggested that the conformational changes of the cytoplasmic PAS domain of DcuS at the oligomeric level are essential for the kinase activation. It is thus possible that although different structural elements are involved in signal transduction, changes at the tertiary structural level, either through conformational changes and/or oligomeric transitions, are used in signal transductions.

Our data using the combined point mutations of the two signalling elements show that the functional state of DctB is determined by a combined net effect of the peri- and cytoplasmic domains. Signal perceived at the ligand-binding site is first transduced by the periplasmic coiled-coil region consisting of L71 and A74, and further transduced via the transmembrane helix and subsequently the S-helix linker containing T366. Our results show that the activities of the hyperactive ‘ON’ mutation in the periplasmic linker are dampened down by the inactive mutant in the S-helix. Likewise, the reduced activity of the ‘OFF’ mutant in the periplasmic linker is to some extent restored by the hyperactive ‘ON’ mutant in the S-helix (figure 7). The effects of individual modules/domains therefore do not dominate, but are instead combined to give rise to the final signal (figure 7*a*). Indeed, our data are in agreement with the two linker regions acting as two transducers/amplifiers, signals perceived by the periplasmic PAS core being thus processed by the these two components, possibly through a dimer to monomer transition and/or tertiary structural changes at the dimer interface, to activate DctB cytoplasmic kinase domains.

Interestingly, although mutations could reduce the induced activities or increase both non-induced and induced activities (table 1), the inducibility (responsiveness) is almost invariantly reduced with the exceptions of L67A, L71I and L71V, suggesting that DctB has a relatively high level of sensitivity to succinate signals. The addition of reducing agent DTT has increased the inducibility of both the wild-type DctB and its L71C mutant, however the inducibility between these two is still similar (that of DctB-L71C is about 20% higher than that of DctB wild-type in the same condition, see table 1). Once the delicate balance of the transduction elements has been affected by modifications (such as L71A, T366R, etc.), the responsiveness to succinate is affected, highlighting its importance in the response to signals. Although the two signal transducers discussed here could amplify or dampen signals and the final signal can be modulated by balancing these two components, the responsiveness to signal appears to be sensitive to mutations. It is thus tentative to suggest that the delicate balance of the system has evolved to respond to specific environmental signals. Considering that HKs are conserved in modularized architecture, usually containing a sensory domain and a conserved cytoplasmic linker, it is possible that this highly specific and sensitive amplifier model could apply to other HKs to generate a system that responds to specific environmental stimuli.

## 5. Material and methods

### 5.1. Domain architecture of DctB and structural analysis of DctBp

The domain architectures of the entire DctB were determined according to the previous studies [7,13] combined with the bioinformatics analysis from SMART (http://smart.embl-heidelberg.de/), TMHMM 2.0 (http://www.cbs.dtu.dk/services/TMHMM/) and Pfam (http://www.sanger.ac.uk/resources/databases/pfam.html). The crystal structures of *S. meliloti* DctBp (PDB ID: 3E4Q for the *apo* state, PDB ID: 3E4O for the succinate-bound state) were used for structural analysis using Pymol (http://www.pymol.org) and Coot [42]. For analysing the ‘knobs into holes’ interaction, the coordinates of 3E4Q were submitted to SOCKET web server (http://coiledcoils.chm.bris.ac.uk/socket/server.html).

### 5.2. Coiled-coil analysis of DctB S-helix linker

The cytoplasmic linker region of DctB (residues 341–410) was used to search against a non-redundant protein database (NCBI) by iterative position-specific BLAST (PSI-BLAST, http://blast.ncbi.nlm.nih.gov/Blast.cgi). Six iterations identified 250 proteins with significant similarities to the DctB linker with an *E*-value less than 0.001. The conservation pattern of the central region is similar to that of the previously identified S-helix linker [25]. Therefore, the central region of the cytoplasmic linker of DctB (residues 350–395) was further analysed by PSI-BLAST and identified as an S-helix linker. Subsequent sequence alignments were carried out by MAFFT v. 5.0 [43] and Jalview v. 2.5 [44]. Secondary structure predictions of DctB S-helix were performed in Jpred [45]. The coiled-coil analysis of DctB S-helix linker was carried out by COILS [34], Paircoil2 [33] and MARCOIL [46].

### 5.3. Genetic manipulations

All constructs for *in vivo* activity assays are based on site-directed mutagenesis of pKU4021 (table 2). For constructs with a single mutation, site-directed mutagenesis was based on the wild-type *dctABD* operon construct generated by PCR that incorporated the appropriate mismatches. For constructs with double mutations, site-directed mutagenesis was based on the mutated *dctABD* operon by PCR with appropriate mismatches. For *in vitro* analysis of disulfide bridges, the *dctB* fragment containing amino acids 1–395 of DctB was cloned and inserted into pBAD-myc/HisA with digesting sites *Bgl*II and *Hin*dIII. All constructs were confirmed by sequencing (BGI, Beijing, People's Republic of China). Detailed information about constructs and strains used in this work is listed in table 2.

**Table 2.** Strains and plasmids used in this study.

strain/plasmid	relevant feature	source
*E. coli* TOP10	for general cloning	Lab stock
*E. coli* TP2339	for p*dctA* driven β-galactosidase activity assay	Lab stock
pSUP102	Wide host range vector	[47]
pBAD/myc–HisA	protein expression vector	Invitrogen
pGD926	*lacZYA* translation fusion vector	[48]
pKU3999	pGD926 with *dctA*::*lacZY*	[7]
pKU4021	pSUP102 with *dctABD*	[7]
pKU4021-L67A	pKU4021 (*dctB^L67A^*)	this study
pKU4021-L71I	pKU4021 (*dctB^L71I^*)	this study
pKU4021-L71V	pKU4021 (*dctB^L71V^*)	this study
pKU4021-L71A	pKU4021 (*dctB^L71A^*)	this study
pKU4021-L71G	pKU4021 (*dctB^L71G^*)	this study
pKU4021-L71C	pKU4021 (*dctB^L71C^*)	this study
pKU4021-L71S	pKU4021 (*dctB^L71S^*)	this study
pKU4021-A74L	pKU4021 (*dctB^A74L^*)	this study
pKU4021-K110D	pKU4021 (*dctB^K110D^*)	[7]
pKU4021-L71A/K110D	pKU4021 (*dctB^L71A/K110D^*)	this study
pKU4021-L71A/A74L	pKU4021 (*dctB^L71A/A74L^*)	this study
pKU4021-T366L	pKU4021 (*dctB^T366L^*)	this study
pKU4021-T366I	pKU4021 (*dctB^T366I^*)	this study
pKU4021-T366V	pKU4021 (*dctB^T366V^*)	this study
pKU4021-T366K	pKU4021 (*dctB^T366K^*)	this study
pKU4021-T366R	pKU4021 (*dctB^T366R^*)	this study
pKU4021-T366D	pKU4021 (*dctB^T366D^*)	this study
pKU4021-T366E	pKU4021 (*dctB^T366E^*)	this study
pKU4021-K110D/T366E	pKU4021 (*dctB^K110D/T366E^*)	this study
pKU4021-L71A/T366L	pKU4021 (*dctB^L71A/T366L^*)	this study
pBAD395	*dctB* (1–395 AA) inserted into pBAD/myc–HisA	this study
pBAD395-L71C	pBAD395 carrying mutation L71C	this study

### 5.4. *In vivo* activity assays for DctB

*In vivo* activity assays of DctB wild-type and mutants were based on previously described procedures [7]. The derivatives of pKU4021 carrying proper *dctB* mutations were transformed into strain TP2339 (*crp-39*, *lacΔX74*) harbouring the reporter pKU3999 (table 2). Single colonies were grown overnight in M63 media (0.4% glucose, 20 mM (NH_4_)_2_SO_4_) at 37°C. The overnight saturated culture was diluted 1 : 200 in the same fresh M63 media in a 37°C shaker until OD_600_ ∼ 0.6. For activity assays with DTT, serial dilutions of DTT were added to the fresh M63 media and the overnight saturated culture were diluted 1 : 100 into the new media. Sodium succinates 2 mM (pH 7.0) was used as the inducer of the *dct* system. β-galactosidase assays were performed according to the method described by Miller [49]. All assays were repeated at least three times. The expression levels of DctB were checked by anti-DctBp antibodies as described previously (electronic supplementary material, figures S1 and S6) [7].

### 5.5. Disulfide bridge analysis

pBAD395 or pBAD395-L71C (table 2) was transformed into *E. coli* strain TOP10. Single colonies were grown overnight in LB media at 37°C. The overnight saturated cultures were diluted 1 : 100 in the fresh LB media in a 37°C shaker until OD_600_ ∼ 0.4. l-Arabinose was added to 5 μM, and the cultures were induced overnight at 18°C. Cultures were harvested by centrifugation at 4°C, and the pellets were resuspended in PBS. DTT 5 mM was added to the sample for about 5 h. Before loading onto the gel, the samples with or without DTT were added with five times loading buffer containing 10% SDS and boiled for 10 min. Denatured samples were loaded on 4–15% gradient SDS-PAGE, and transferred to the Hybond-C Extra PVDF membrane by semi-dry transfer apparatus (Bio-Rad). The membrane was blocked overnight with 10% non-fat milk. The membrane was then probed with 1 : 3000 dilution of anti-His antibody (TianGene) and followed by 1 : 5000 dilution of peroxidase-conjugated goat anti-mouse IgG (H + L) (ZSGB-Bio). The antibody was detected using metal-enhanced DAB Substrate Kit (Thermo) according to the manufacturer's protocol.

## Supplementary Material

Supplemental Figures
