# Supplementary material for: Mutational analysis of dimeric linkers in peri- and cytoplasmic domains of histidine kinase DctB reveals their functional roles in signal transduction
Source: Open Biol. 2014 Jun 4;4(6):140023. doi: 10.1098/rsob.140023 (PMC4077058; doi:10.1098/rsob.140023)
Supplement: Supplemental Figures [file rsob140023supp1.pdf]

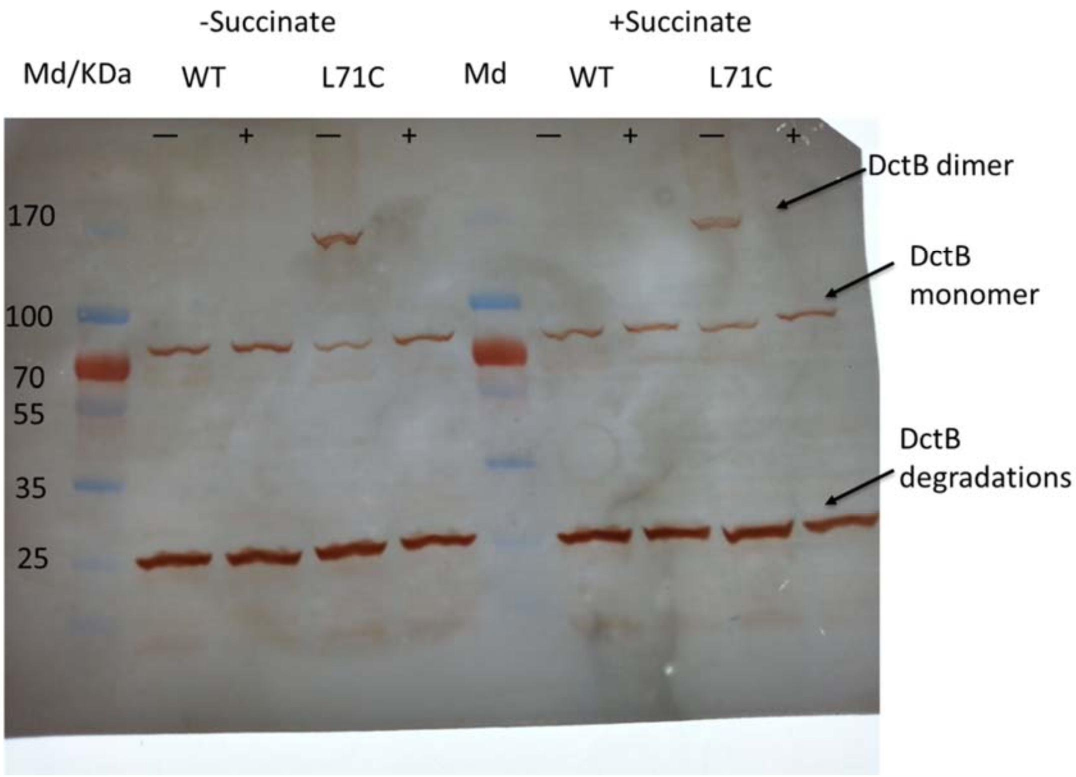

Fig. S1. *In vitro* analysis of disulfide bridge of DctB.

The bacteria used for measuring *in vivo* activities with or without DTT were used. The membrane was probed with anti-DctBp antibody, followed by anti Rabbit IgG (H + L) HRP and stained by Metal Enhanced DAB Substrate Kit (Thermo) according to the standard method.

A

```
                      358 362  366       376
DctB S-Helix  EERLARNALEASVEERTRDLRMARDRLETEIADHRQTTEKLQAVQQ
Paircoil2     cdefgabcdefcdefgabcdefgabcdefgabcdefgabcdefgef
Coiled-Coils  cdefgabcdefgdefgabcdefgabcdefgabcdefgabcdefgab
MARCOIL       cdefgabcdefgdefgabcdefgabcdefgabcdefgabcdecdef
COILS/PCOILS  cdefgabcdefgaefgabcdefgabcdefgabcdefgabcdefgab
MultiCoil     .......cdefgabcdefgabcdefgabc................
```

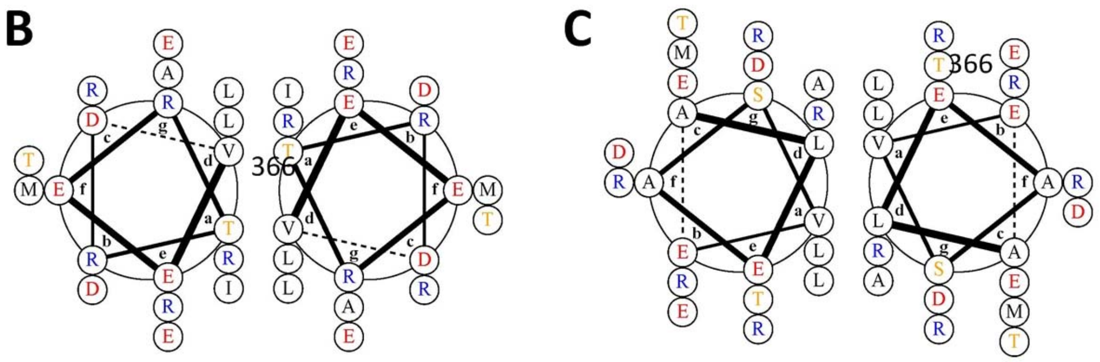

Fig. S2. Coiled-coil analysis of DctB S-Helix linker sequence.

(A) Coiled-coil prediction using different programs. Paircoil2, Coiled-coils, MARCOIL, COILS/PCOILS all predict the existence of a stutter element around residues S361 and V362. The conserved hydrophobic residues and the polar reside (T366) are underlined and their sequence numbers

are marked above.

(B) Projected projection view of the central region of the S-Helix coiled coil (residue V362 ~ I380), using the first four register shown in (A).

(C) Projected projection view of the central region of the S-Helix coiled coil (residue V362 ~ I380), using the prediction by MultiCoil shown in (A).

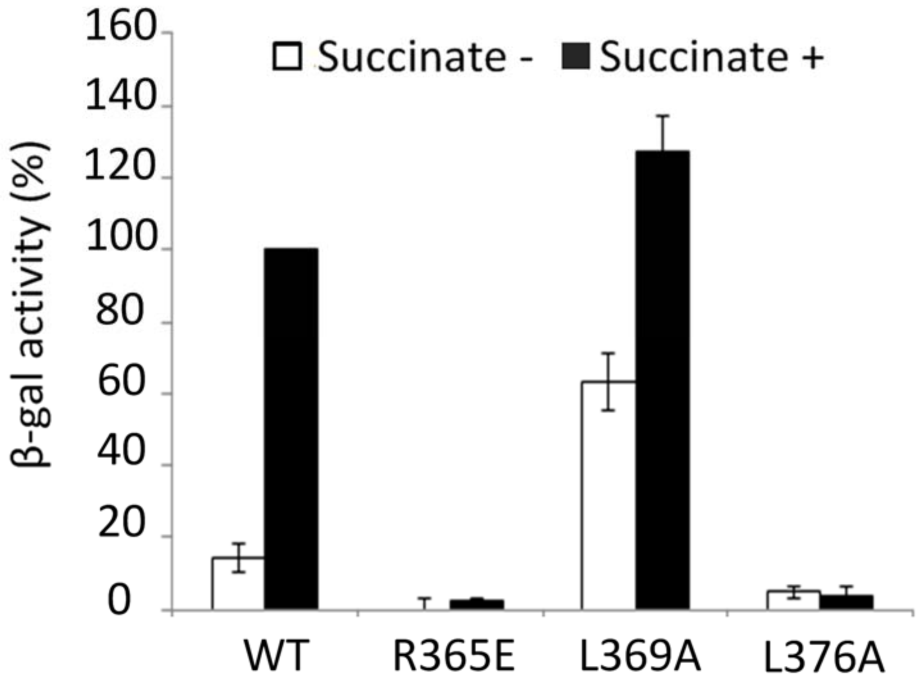

Fig. S3. *In vivo* activities of selective mutations on the S-Helix. All activity assays were performed as described in Fig. 2.

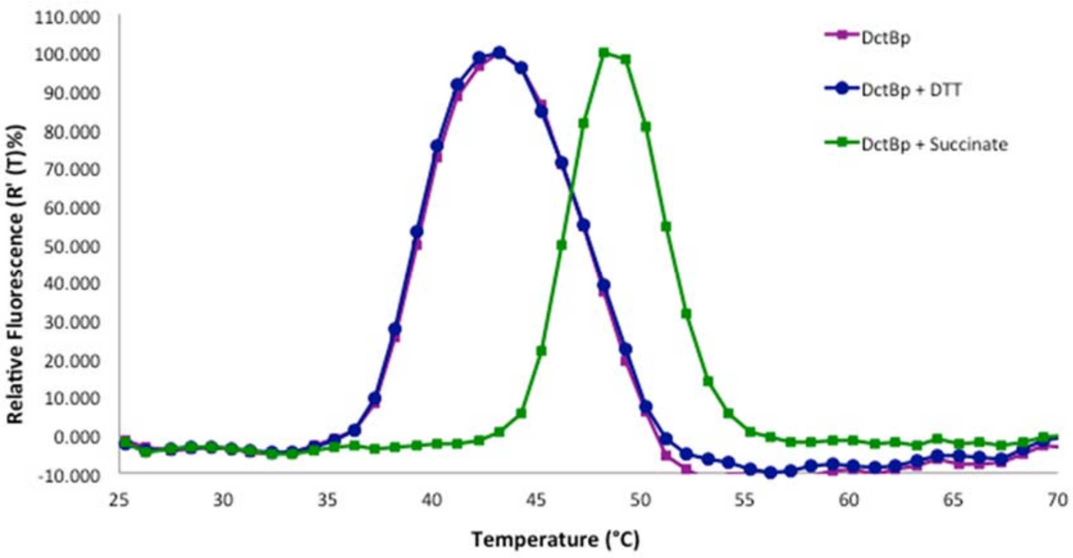

Fig. S4. Melting curves of DctBp.

Melting curves of DctBp (1mg/ml) in 20mM Tris pH8, 200 mM NaCl buffer and 1:1500 diluted SYPRO Orange as the reporter dye were monitored using RT-PCR instrument. 10 mM succinate (pH 7.0) or 10

mM DTT were incubated with the protein for 30 minutes at room temperature before heating. The melting temperature of DctBp shows no changes upon the addition of DTT, instead, about 5 degrees increase upon the addition of succinate, confirming that DctBp binds to succinate but not DTT.

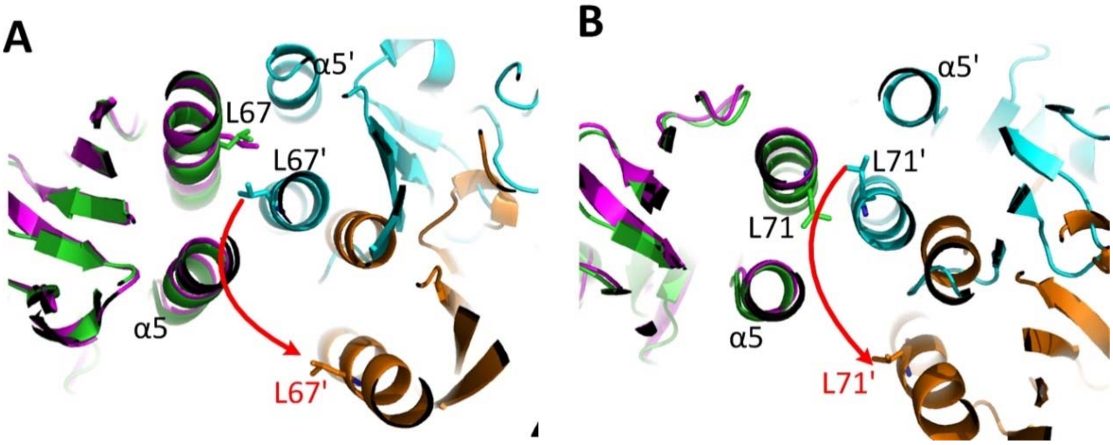

Fig. S5. Structural Comparisons between L67 and L71 residues in apo and succinate-bound states.

(A) L67-L67' are loosely associated on the dimeric interface in both states and one L67 do not "lock " the other during the conformation change upon ligand binding.

(B) The side chains of L71-L71' are "locked" in the apo state, and upon ligand-binding, the two residues need to be "unlocked" to move apart from each other.

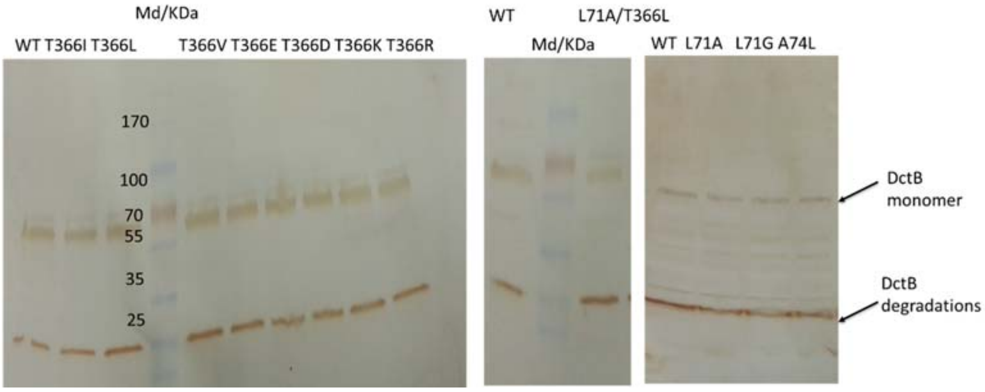

Fig. S6. Expression levels of DctB wild-type and mutant proteins.

The bacteria used for measuring *in vivo* activities were used. The western blots were carried out as in Fig. S1. No significant changes in expression level were observed between DctB wild type and mutants.
